# Charting the path in rodent functional neuroimaging

**DOI:** 10.1162/IMAG.a.12

**Published:** 2025-05-28

**Authors:** Alessandro Gozzi, Alexia Stuefer, Filomena Grazia Alvino, Valeria Bedin, Christopher Cover, Chaewon Kang, Alberto Galbusera, Rita Gil, Silvia Gini, Elizabeth de Guzman, Gabriel Desrosiers-Grégoire, Daniel Gutierrez-Barragan, Francesca Mandino, Jean-Charles Mariani, Edoardo Micotti, Henning Reimann, Marco Pagani, Chiara Pepe, David Sastre-Yagüe, Mila Urosevic, Mafalda Valente, Roberta Vertullo, Rossella Canese, Anna Devor, Joanes Grandjean, Itamar Kahn, Shella D. Keiholz, Evelyn M.R. Lake, Nan Li, Noam Shemesh, Yen-Yu Ian Shih, Valerio Zerbi, Nanyin Zhang

**Affiliations:** Functional Neuroimaging Laboratory, Center for Neuroscience and Cognitive Systems, Istituto Italiano di Tecnologia, Rovereto, Italy; Center for Mind and Brain Sciences, University of Trento, Rovereto, Italy; Department of Bioengineering, University of Pittsburgh, Pittsburgh, PA, United States; Champalimaud Research, Champalimaud Foundation, Lisbon, Portugal; Computational Brain Anatomy Laboratory, Cerebral Imaging Center, Douglas Mental Health University Institute, Montreal, Canada; Integrated Program in Neuroscience, McGill University, Montreal, Canada; Department of Radiology and Biomedical Imaging, Yale School of Medicine, New Haven, CT, United States; Istituto di Ricerche Farmacologiche Mario Negri IRCCS, Milano, Italy; Berlin Ultrahigh Field Facility (B.U.F.F.), Max-Delbrück Center for Molecular Medicine, Helmholtz Association of German Research Centers (HZ), Berlin, Germany; Autism Center, Child Mind Institute, New York, NY, United States; IMT School for Advanced Studies, Lucca, Italy; Istituto Superiore di Sanità, Rome, Italy; Department of Biomedical Engineering, Boston University, Boston, MA, United States; Donders Institute for Brain, Behaviour, and Cognition, Radboud University, Nijmegen, The Netherlands; Department for Medical Imaging, Radboud University Medical Center, Nijmegen, The Netherlands; Department of Neuroscience and Zuckerman Mind Brain Behavior Institute, Columbia University, New York, NY, United States; Department of Biomedical Engineering, Georgia Institute of Technology and Emory University, Atlanta, GA, United States; Department of Biomedical Engineering, Yale University, New Haven, CT, United States; Advanced Imaging Research Center, UT Southwestern Medical Center, Dallas, TX, United States; Center for Animal MRI, School of Medicine, University of North Carolina at Chapel Hill, Chapel Hill, NC, United States; Biomedical Research Imaging Center, School of Medicine, University of North Carolina at Chapel Hill, Chapel Hill, NC, United States; Department of Neurology, University of North Carolina at Chapel Hill, Chapel Hill, NC, United States; Department of Biomedical Engineering, University of North Carolina at Chapel Hill, Chapel Hill, NC, United States; Department of Psychiatry, Faculty of Medicine, University of Geneva, Geneva, Switzerland; Department of Basic Neurosciences, Faculty of Medicine, University of Geneva, Geneva, Switzerland; Department of Biomedical Engineering, The Pennsylvania State University, University Park, PA, United States; Center for Neural Engineering, The Pennsylvania State University, University Park, PA, United States

**Keywords:** mouse, fMRI, awake, fUSI, anesthesia, rat

## Abstract

Driven by a period of accelerated progress and recent technical breakthroughs, whole-brain functional neuroimaging in rodents offers exciting new possibilities for addressing basic questions about brain function and its alterations. In response to lessons learned from the human neuroimaging community, leading scientists and researchers in the field convened to address existing barriers and outline ambitious goals for the future. This article captures these discussions, highlighting a shared vision to advance rodent functional neuroimaging into an era of increased impact.

## 1 Introduction

Over the past decade, rodent whole-brain functional neuroimaging, particularly through functional magnetic resonance imaging (fMRI), has made significant progress in addressing both basic and applied research questions on brain function. Much of this progress has been driven by the expansion of rodent fMRI’s reach to include large-scale mapping of intrinsic brain activity, often referred to as “functional connectivity”, or “fMRI connectivity” (Gozzi & Schwarz, 2016). As part of this research, the combined use of rodent fMRI with genetic modeling (Gozzi & Zerbi, 2023), chemo-optogenetics (Kim et al., 2023;Mandino et al., 2022;Menon et al., 2023;Oyarzabal et al., 2022;Rocchi et al., 2022;Wang et al., 2023;Yu et al., 2016;Zerbi et al., 2019), or advanced multimodal recordings (Cabral et al., 2023;Jung et al., 2021) has enabled the exploration of the neural mechanisms underlying brain network activity, fMRI connectivity, and behavior (Gil et al., 2024) with remarkable precision. Importantly, the discovery of plausible rodent precursors of human fMRI connectivity networks (Pagani et al., 2023;Sforazzini et al., 2014) has made it possible to directly translate preclinical findings to human research, revealing very encouraging cross-species correspondences (Bertero et al., 2018;Gutierrez-Barragan et al., 2024;Mandino et al., 2022;Pagani et al., 2023). Overall, rodent functional neuroimaging is strategically positioned to address several long-standing questions in modern systems neuroscience, while also facilitating the direct cross-species translation of results in biomedicine. However, despite the rapid growth and success of this approach, its implementation is not without inherent challenges. Recently, leaders in the field of rodent fMRI gathered in Rovereto, Italy, to address the unique challenges facing this area of research and to identify collaborative solutions aimed at accelerating progress over the next 5–10 years. Three macro areas of key intervention were identified that could benefit from community-fostered solutions: the adoption of awake functional brain mapping, the implementation of multimodal neuroimaging, and the standardization of data acquisition and analysis.

## 2 Towards Awake Rodent Neuroimaging

Rodent functional neuroimaging relies overwhelmingly on controlled sedation. This approach has become the gold standard in rodent fMRI owing to its ease of implementation as well as the possibility of tightly controlling restraint-induced stress and imaging artifacts caused by head and/or body motion. However, a wide array of anesthetic protocols have been adopted across small-animal fMRI labs, hindering cross-lab comparisons. Moreover, sedatives can unpredictably interact with ongoing neural and hemodynamic activity, thus biasing the distribution and organization of resting and evoked fMRI activity (Hamada et al., 2024). The use of sedatives also restricts brain states and prevents the investigation of task-activated networks.

Recent attempts to avoid the use of anesthesia have shown promise, documenting the possibility to reliably detect fMRI activity in non-anesthetized animals habituated to the scanner (Desai et al., 2011;Gutierrez-Barragan et al., 2019;Liang et al., 2011). However, despite this initial progress, technical and interpretive challenges greatly complicate the implementation of awake rodent imaging (discussed below). For this reason, the use of controlled sedation remains a valuable experimental option that can crucially integrate and complement ongoing efforts toward the implementation of awake functional neuroimaging.

### 2.1 Controlled sedation is here to stay

There is a general agreement that controlled sedation continues to hold significance and utility due to several unique advantages. These include, for example, tight control of arousal levels and brain state, an essential requisite in chemo/optomodulation studies (Giorgi et al., 2017;Grimm et al., 2024), or in the use of peripheral sensory stimulation, in which a prolonged modulation of brain activity under quasi-stable states is highly advantageous. The use of anesthesia also plays a critical role in minimizing motion and related artifacts, resulting in higher quality data and a considerably faster and less resource intensive turnout. While sedation may influence the functional organization of certain brain networks and their relation to behavior, it also effectively eliminates the need for prolonged immobilization, and avoids potential functional impacts from repeated acclimation training. Moreover, the functional organization of brain networks appears to be largely preserved by multiple sedation regimens, resulting in functional networks that are broadly comparable to those obtained from awake animals. Furthermore, neuroimaging with anesthesia preparation in rodents remains a key avenue of investigating systems-level mechanisms underlying anesthesia-induced unconsciousness (X. Chen et al., 2024).

Notwithstanding its convenience, the use of anesthesia has its challenges. Conducting neuroimaging experiments under controlled sedation requires extremely careful consideration of the specific anesthetic mechanism employed, the desired anesthesia depth, and experimenters should be mindful of possible unpredictable interactions between anesthesia and the stimulation employed (Hamada et al., 2024) or the (patho)physiology being investigated. For example, the anesthetic states produced by light or deep isoflurane anesthesia are very different, and commonly used gaseous halogenated anesthesia tends to accumulate in fat tissue, potentially altering anesthetic state (Gozzi et al., 2008;Sirmpilatze et al., 2022). Similarly, injectable anesthetics are also characterized by evolving pharmacological efficacy over time (Nasrallah et al., 2014). Thus, tight control and standardization of anesthesia depth are crucial in experiments comparing groups or conditions. Without this, large spurious changes in resting or evoked fMRI activity due to variations in anesthesia depth may obscure or worse, be misinterpreted as, biologically plausible effects.

In the case of experiments encompassing transgenic lines (for example, mutant versus wildtype animals), genotype-dependent differences in sensitivity to anesthesia can be probed via measurements of minimal alveolar concentration (Sonner et al., 2000). However, if differences in anesthetic susceptibility between models arise, it remains unclear how these should be dealt with. More broadly, the implementation of measurements allowing for a standardization of anesthesia depth in individual animals is of crucial importance. Anecdotally, intubation and artificial ventilation provide tighter control of anesthesia depth compared to standard nose cones and allow a more stable control of blood gas levels, although this procedure significantly increases experimental complexity (Ferrari et al., 2012). Alternatively, adjustment of anesthesia levels to reach a target respiratory frequency, or a detectable response to exogenous visual or sensory stimulation, can be used to harmonize anesthesia depth across animals (Oyarzabal et al., 2022). We, however, note that, at present, no universal quality control measure for anesthesia depth is available. Future efforts are required to establish these metrics and compile a set of best sedation practices, validated across different research sites.

To complicate matters, peripheral measurements of physiological parameters that can directly or indirectly fMRI BOLD activity, like arterial blood gas levels, or blood pressure, are technically challenging, and present species-specific constraints. For example, arterial pCO2 can be non-invasively recorded in rats through the use of transcutaneous monitors (Ramos-Cabrer et al., 2005). However, this method does not appear to be reliable in mice. Similarly, arterial blood pressure requires surgical femoral artery cannulation, a procedure that is technically challenging in mice, where it requires specialized skills (Ferrari et al., 2012). To mitigate physiological variability and improve data quality, laboratories have so far employed non-invasive monitoring tools for heart rate, respiratory rate, and oxygen saturation via pulse-oxymetry. However, newcomers to the field should be aware that the implementation of these measurements is more challenging and less reliable in smaller animal species.

Researchers should also be aware that anesthetic agents may produce largely different brain states as a function of their pharmacological profile. For example, medetomidine produces waves and spindles reminiscent of NREM sleep, low-level halothane anesthesia mostly consists of high-frequency power (similar to REM sleep), while ketamine produces wake-like EEG (Gent & Adamantidis, 2017;Reimann & Niendorf, 2020). In this respect, the widespread reliance on a medetomidine-isoflurane combination in rodent studies, while useful for comparing conditions across laboratories, has the drawback of limiting fMRI investigations to a single, stereotypical electrophysiological state. Efforts should, thus be directed at widening the current repertoire of sedative regimen. More importantly, given the varying interactions that different anesthetic mechanisms can exert with different neuromanipulations, it is of paramount importance to assess how the resulting functional effects may generalize across different anesthetic regimens, and potentially to awake conditions via proof-of-concept awake studies (Gutierrez-Barragan et al., 2022;Rocchi et al., 2022).

While the possibility of reliably mapping functional connectivity networks with fMRI represents one of the most notable recent extensions of functional neuroimaging in rodents, the translational impact of this approach is not limited to resting paradigms. Sensory and opto-chemogenetic stimulations are increasingly used to uncover the neural basis of neurovascular coupling and to visualize central pathways implicated in sensory processing, with important implications for our understanding of how neural activity is translated into hemodynamic signals (Cerri et al., 2024;Gil et al., 2024)*.*As with studies conducted at rest, a number of unresolved technical questions accompany functional stimulation studies in rodents. Key among them is the unclear influence of various anesthetic mixtures on sensory-evoked responses (Rungta et al., 2017), as well as the yet undetermined contribution of local versus nonspecific hemodynamic responses to sensory stimuli (Pisauro et al., 2016). Additionally, the confounding role of peripheral cardiovascular factors, such as stimulus-induced blood pressure changes, remains under-investigated (Gozzi et al., 2007;Reimann et al., 2018). Community efforts toward paradigm standardization and benchmarking are critically required also in this area of investigation, with initial results showing promise (Grandjean et al., 2020,^2023^).

### 2.2 Navigating the complexity of awake functional neuroimaging

Despite the convenience of anesthesia, a transition to awake imaging is a desired evolution of functional brain mapping in small animals. The opportunities offered by reliable awake functional interactions between anesthesia and different kinds of neuromanipulations, to the lack of unwanted interferences with neurovascular coupling, up to a more direct translation of fMRI findings to neuroscience methods used in awake rodents, such as calcium imaging or electrophysiology.

While convincing examples of fMRI mapping in awake rodents have been demonstrated in the past few years (Bergmann et al., 2016;Gutierrez-Barragan et al., 2022;Liang et al., 2012), the implementation of awake imaging comes with a number of foundational challenges. One of the main hurdles is animal immobilization, which is typically achieved through head-fixation or tight body restraint procedures. This process often requires surgical apposition of custom implants and time-consuming habituation to reduce stress from prolonged immobility. Additionally, the scanner environment presents its own set of stressors, including noise and vibrations from gradient operations, the novelty of the scanner, and the presence of odors left by other stressed conspecifics, predators, or reagents due to the shared use of the scanner by multiple research teams.

To date, the effect of different habituation procedures on image quality and arousal state of animals remains unclear (reviewed inMandino et al. (2024)). The duration of habituation protocols varies widely across studies, typically ranging from 6 to 14 days. A conservative guideline suggests a period of 10–14 days for robust acclimation in a conventional fMRI setting (Mandino et al., 2024). However, shorter durations (6 to 8 days) may still be effective, particularly when key elements, such as exposure to the real scanner, are incorporated (Dinh et al., 2021;Zou et al., 2024). A critical challenge in evaluating acclimation efficacy is the inconsistent use of stress hormone measurements. These measurements are difficult to obtain using non-invasive approaches, and are further complicated by methodological discrepancies in sample collection and the absence of established reference levels in the literature. Notwithstanding these issues, acclimation procedures have been convincingly shown to substantially reduce corticosterone levels across different laboratories (Mandino et al., 2024)). This suggests that acclimation may be effective in reducing stress. However, the extent to which this reduction compares to non-restrained or freely behaving conditions remains unclear. Moreover, while habituation can effectively reduce stress, the demands of high-throughput environments where efficiency is key (such as in pharmaceutical research) can present logistical challenges. Strategies such as exposure to mock scanners and batch training can improve the feasibility of awake fMRI in these settings, but acclimation procedures remain labor-intensive and difficult to implement on a large scale.

To address these challenges, we propose three complementary approaches. First, benchmark cross-laboratory studies are required to foster standardization and to more rigorously assess the effectiveness of habituation procedures on stress and image quality. This could follow the model of a recent initiative that established a community protocol for anesthetized rat fMRI (Grandjean et al., 2023). Second, the development and optimization of free induction decay (FID)-based MRI sequences (Idiyatullin et al., 2015;Ljungberg et al., 2021;Madio & Lowe, 1995;Weiger & Pruessmann, 2019) for fMRI applications should be prioritized. Initial evidence of the convenience of this approach has been demonstrated using multi-band sweep imaging with Fourier transformation (MB-SWIFT) (Dvořáková et al., 2024;Lehto et al., 2017;Paasonen et al., 2020), followed by zero echo time (ZTE), and Steady-state On-the-Ramp Detection of INduction-decay with Oversampling (SORDINO, (MacKinnon et al., 2025) sequences. These techniques offer promising alternatives to conventional Echo-planar imaging (EPI)-based BOLD fMRI, addressing key challenges such as acoustic noise, susceptibility artifacts, and motion-related confounds, which are critical factors in awake rodent fMRI. We anticipate broader adoption of these techniques in the coming years. However, these alternative fMRI sequences face their own challenges, including less-well understood contrast mechanisms compared to BOLD, limitations in sequence and reconstruction generalizability, and constraints in imaging resolution and sensitivity. Moreover, because EPI remains the most widely used fMRI method in humans, understanding the physiological basis of EPI-based fMRI signals will remain a focus of rodent functional neuroimaging. Finally, precise in-scanner control of rodent behavior and physiology is essential. This may involve the development, temporal alignment, and standardization of sensors to monitor a range of behaviorally and physiologically relevant variables, including head motion, breathing, facial expressions, and pupil size (Hike et al., 2024).

A more fundamental challenge related to awake whole-brain functional neuroimaging lies in determining the significance of the actual brain states achieved in non-compliant, scanner-habituated animals like rodents, which are known for engaging in almost continuous behavioral activity (like sniffing, whisking, etc. (Winder et al., 2017)). When applied to the implementation of resting-state fMRI, the question that arises is thus whether rodents are ever truly “at rest”. This problem does not pertain to fMRI specifically. A number of influential electrophysiological or optical imaging studies have, indeed, shown that awake head-fixed mice continually engage in overt (for example, locomotor activity), as well as more nuanced volitional behaviors and motion outputs (like whisking, or facial movements, etc.) (Stringer et al., 2019;Winder et al., 2017). The issue at hand does not solely pertain to the adverse effects of motion on signal quality. Rather, it encompasses the broader possibility that the resulting functional signals may significantly, or even primarily, reflect ongoing behavioral or motor outputs. To address this issue, recent investigations of “resting brain activity” in awake head-fixed mice have encompassed the concatenation of calm periods among motile bouts (Bertolo et al., 2023). However, this approach limits usable recordings to only a small fraction of the acquired time series. Complicating matters further, behavioral and motion outputs in head-fixed mice tightly covary with peripheral indexes of arousal such as pupil diameter and explain infraslow dynamics in neural signals relevant to the interpretation of resting-state fMRI (Stringer et al., 2019). Moreover, neurovascular coupling appears to evolve as a function of arousal state (Meyer-Baese et al., 2024).

A pressing question that the field of awake neuroimaging must address is whether and how the ever-evolving arousal state of awake head fixed rodents, including activity recorded in movement-free bouts, compare to the “quiet wakefulness” that characterizes most human fMRI studies. In this respect, the possibility that scanner habituated animals are in a higher arousal state than human counterparts should always be considered when interpreting the results of rodent neuroimaging studies in the context of translational research. Furthermore, high-arousal states may confound stimulation studies (like those using optogenetics) by eliciting unintended motor or interoceptive signals that might mask or confound those generated at the primary stimulation site. Overall, a trans-disciplinary understanding of the impact of arousal and volitional movement on recordings of “resting” brain activity is a key priority of functional neuroimaging in small animals. While we await to obtain a deeper understanding of this phenomenon, researchers transitioning to awake imaging should recognize and account for this central problem.

The implementation of sensory stimulations or task-based fMRI in awake, behaving rodents is at present highly problematic, mostly a consequence of high susceptibility of fMRI to apparent (B0-related) motion-induced artifacts, and the constraints related to the limited available space within the scanner bore. We expect advances in FID-based, center-out encoding fMRI techniques (Dvořáková et al., 2024;Paasonen et al., 2020) to facilitate this endeavor by minimizing acoustic noise interference and susceptibility to motion artifacts, thus enabling the implementation of more complex tasks (MacKinnon et al., 2025). There is consensus in the field that stimulation- and task-based functional neuroimaging are highly complementary to resting-state mapping, and both should be pursued to enhance the translational impact of small-animal functional neuroimaging. Prompted by the limitations of stimulation or task-based fMRI in awake animals, a growing portion of the imaging community is now considering alternative neuroimaging methods to relate large-scale activity to behavior. In this context, functional ultrasound imaging (fUSI) has shown the greatest promise, owing to its excellent portability and its ability to record whole-brain patterns of functional activity during complex behavioral tasks (El Hady et al., 2024). Although still in its infancy, fUSI also offers a notable advantage over fMRI: its substantially lower cost. This is particularly relevant in the context of preclinical MRI systems, where a monopolized market (Freeman & Morris, 2015) has led to steadily rising operational costs over the past two decades. As a result, there is a genuine concern that preclinical MRI may become economically unsustainable, even for well-funded laboratories and institutions. The preclinical community, with the support of their hosting institutions, should unite in lobbying leading MRI scanner manufacturers to make them aware of these concerns, and to ensure that this technology remains broadly accessible and economically sustainable.

### 2.3 Towards multimodal functional neuroimaging

#### 2.3.1 Chemo- and optogenetic-fMRI

One of the most important contributions of rodent functional neuroimaging to the broader neuroscience community is the ability to probe how cell-type specific manipulations can affect large-scale patterns of brain activity. The combination of fMRI with chemogenetics or optogenetics (chemo-fMRI (Giorgi et al., 2017), opto-fMRI (Kahn et al., 2013;J. H. Lee et al., 2010)) represents an investigational platform that is unique in its ability to probe causality between neural events at different investigational scales. Importantly, the causal nature of these approaches serves as a key mechanistic complement to the inherently correlational neuroimaging approaches available in humans (Pagani et al., 2023;Rocchi et al., 2022;Siddiqi et al., 2022).

Chemo- and optogenetic-fMRI in rodents have become increasingly prominent over the past few years (Bernal-Casas et al., 2017;Cerri et al., 2024;Chan et al., 2017;X. Chen et al., 2019;Y. Chen et al., 2020;Choe et al., 2018;Christie et al., 2013;Cover et al., 2021;Decot et al., 2017;Desai et al., 2011;Ferenczi et al., 2016;Giorgi et al., 2017;Gozzi et al., 2010;Grandjean et al., 2019;Grimm et al., 2021;Hamada et al., 2024;Han et al., 2022;Helbing et al., 2024;Ioanas et al., 2022;Iordanova et al., 2015;Jung et al., 2021,^2022^;Kahn et al., 2011,^2013^;Kargl et al., 2020;Kim et al., 2023;J.H. Lee et al., 2010,H. J. Lee et al., 2016;J. Y. Lee et al., 2022;Leong et al., 2016,^2021^;N. Li et al., 2011;Z. Li et al., 2023;Y. Li et al., 2024;Liang et al., 2015;Mandino et al., 2024;Markicevic et al., 2023;Menon et al., 2023;Moon et al., 2021;Nakamura et al., 2020;Oyarzabal et al., 2022;Peeters et al., 2020;Rocchi et al., 2022;Roelofs et al., 2017;Ryali et al., 2016;Salvan et al., 2021;Sanda et al., 2024;Schmid et al., 2017;Takata et al., 2018;Tu & Zhang, 2022;Vo et al., 2023;Wang et al., 2023;Wei et al., 2021;Weitz et al., 2015;Yu et al., 2016;Zerbi et al., 2019;Zou et al., 2024). Notable examples of the application of these methods include the possibility of non-invasively dissecting large-scale networks and circuits via targeted optogenetic stimulations and inhibitions (Jung et al., 2022;J. Y. Lee et al., 2022). The application of this paradigm to sensory stimulations can help identify dysfunctional circuits and determine potential therapeutic targets in disease models (J. Y. Lee et al., 2022;Lee et al., 2024). The growing utilization of these methods has enhanced our ability to identify and control for technical limitations and confounding artifacts related to their implementation, including heat- or visually-induced artifacts from light stimulation (Schmid et al., 2017), as well as off-target hemodynamic effects produced by commonly used chemogenetic ligands (Giorgi et al., 2017;Zerbi et al., 2019). As these approaches become more accessible, future research needs to be directed at determining how these manipulations affect and interact with different brain states, both in anesthetized and awake conditions, and how these are impacted by peripheral confounds produced by these manipulations. In this respect, the implementation of surrogate measures of arousal, such as pupillometry, represents a key extension to chemo- and opto-fMRI recordings. Opportunities also exist for introducing standardized protocols and experimental designs such that the physiological signals and peripheral effects elicited by the chemo- and optogenetic manipulations can be benchmarked across laboratories. These investigations should include measurements of respiratory and peripheral cardiovascular parameters such as heart rate. We also note here that in both chemo- and opto-fMRI studies, the role of stimulus-evoked arterial blood pressure changes has been for the most part neglected, despite its established confounding contribution to central hemodynamic measures of brain activity (Ferrari et al., 2012). More importantly, it is also crucial that the field comes to a general agreement about the proper design and validation of chemogenetic-fMRI. A major confounding factor is the lack of electrophysiological validation of target chemogenetic manipulations in the specific brain area, and under the specific experimental conditions of the fMRI experiment. Such validation is a necessary prerequisite for interpreting chemo-fMRI results, given the highly variable effects chemogenetic effectors can produce across brain regions, species, and promoters (Kovács et al., 2024;Rocchi et al., 2022). We also note that opto-fMRI cannot be straightforwardly implemented with standard radiofrequency cryorefrigerated coils, because of limited space. There is, thus, a need for advanced customized setups and/or fiber designs that can be used in conjunction with cryocoil technology (Grimm et al., 2022;Zou et al., 2024).

#### 2.3.2 Multimodal recordings of brain activity

Multimodal recordings of both spontaneous and stimulus-evoked fMRI activity can critically advance our elusive understanding of these phenomena both at the mesoscopic (Rungta et al., 2017) and large-scale level. This area of investigation is of strategic importance given the widespread use of fMRI to indirectly infer neural activity in human cognitive neuroscience both in task-evoked and in task-free settings.

Notable examples of the use of small-animal fMRI to uncover the neurovascular cascade underlying the BOLD response have been reported in recent years via the combination of multimodal optical and/or electrophysiological recordings (Cerri et al., 2024;Vo et al., 2023). The initial extension of these approaches to investigate the underpinnings of large-scale fMRI coupling has also been recently described (Rocchi et al., 2022;Tu & Zhang, 2022). In this respect, emerging hybrid imaging technologies such as PET/fMRI hold great promise, as they offer the unique capability to enhance fMRI with metabolic and molecular insight (Wehrl et al., 2013). While PET/MRI has not yet been extensively applied to investigate the bases of fMRI connectivity in rodents, this approach could help clarify the relationship between neuronal activity and specific metabolic or molecular processes, including the contribution of intrinsic neurotransmitter activity (Haas et al., 2024;Ionescu et al., 2025;Millevert et al., 2023). Similarly, multimodal fMRI may now uniquely leverage progress in the miniaturization of electrophysiological probes, and the rapidly increasing repertoire of fluorescent encoders (Sabatini & Tian, 2020) to provide an unprecedented multifaceted dissection of the neural and hemodynamic processes underlying fMRI. Recent technological advancements also include the combination of fMRI with widefield optical imaging, an approach that may uniquely cross-link neural and hemodynamic signals at the meso- and macroscale (Lake et al., 2020).

As promising as these approaches are, the entrance barrier for these methods in terms of cost, equipment, and technical skills required for data integration is still substantial. While the level of complexity of electrophysiological and optical setup differs significantly, the implementation of multimodal fMRI still relies on the use of customized parts and apparatuses that required dedicated know-how, and that only the best-equipped labs are in a position to implement via dedicated personnel or workshop units. The analysis of the ensuing signals and their denoising (like gradient filtering in electrophysiology, hemodynamic correction in calcium imaging, co-registration in widefield imaging) are also a complex endeavor that at present does not seem to be readily exportable across labs. Given the pioneering stage of this research area and the lack of standardized solutions, the preclinical community should aim to promptly share relevant information (including design blueprints, guidelines, code, and procedures) in open repositories to facilitate replication by capable laboratories. The organization of theoretical and hands-on training courses, possibly covering other areas of functional neuroimaging, would also greatly boost the adoption of these methods. To drive this forward, we need to establish a growing community base that includes all major contributors in the field (including the many who were not part of this initial impromptu working group), to create a larger and more authoritative network to guide progress and standardization.

### 2.4 Unifying standards for data acquisition and analysis

#### 2.4.1 Data acquisition protocols

As emphasized throughout this manuscript, advancing rodent functional neuroimaging requires a concerted effort toward the standardization of procedures. The recent adoption of common data curation and sharing standards (for example, Brain Imaging Data Structure – BIDS (Gorgolewski et al., 2016)) paves the way for similar standardization efforts at the data acquisition and experimental design stage.

All the participants in this working group are familiar with multiple instances of inadequately labeled datasets and scantily described experimental methods. To address this, we propose establishing a consensus on minimal data and metadata descriptors (including MRI coils, sequences, animal cradle design) and developing software tools to automate data curation, including parsing metadata to organize datasets into the BIDS format. We also advocate strict adherence to ARRIVE guidelines for animal experiment reporting (Percie du Sert et al., 2020). By improving reporting practices and identifying a rational set of optimal parameters through cross-laboratory initiatives (Grandjean et al., 2020), we anticipate that the field will naturally transition toward consensus on a set of benchmarked experimental procedures.

The advantages of a transition toward the use of standardized data acquisition protocols are substantial. Firstly, standardization can greatly facilitate transfer of know-how, and comparison of results via a cross-laboratory data benchmarking based on existing literature. Standardized protocols also allow for published datasets to be aggregated into multi-center studies to increase statistical power (Zerbi et al., 2021). By minimizing the need for extensive piloting, standardized data acquisition procedures can also significantly reduce the workload for experimenters. Furthermore, consensus protocols can help increase confidence in reported findings, as reviewers and readers can compare baseline metrics with previously reported values to verify data quality. Finally, standardized data acquisitions may facilitate the development of relevant software and robust exploratory pipelines.

Notwithstanding the virtues of standardized protocols, this workgroup acknowledges that “standardization is a tool, not a policy”. As such, the application of consensus protocols is not meant to be dogmatic, nor should it constrain the experimental needs or creativity of researchers. Standardization guidelines should rather be viewed as recommendations, particularly in instances where no rational basis favors one particular set of parameters. They may also serve as helpful directives during the initial stages of projects. Additionally, standards should not supplant pilot studies, the outcomes of which are encouraged to be published as supplementary materials.

#### 2.4.2 Analysis pipelines

There is wide consensus among investigators of this working group on the importance of developing end-to-end, user-friendly, and open-source preprocessing pipelines tailored for rodent functional neuroimaging analysis. So far, the field has mostly relied on in-house implementations built on processing tools developed for human neuroimaging (like AFNI, FSL, etc.). However, these analytic tools are not always directly back translatable to rodent fMRI data and, as such, require workarounds to be used.

While most investigators agree that a minimal processing approach might be sufficient for rodent functional neuroimaging data (Grandjean et al., 2020,^2023^), establishing empirical, data-driven quality metrics could significantly enhance the reliability of data quality assessments across laboratories. Standardizing pipelines and quality assurance metrics will not only improve the rigor and reproducibility of findings, but also encourage researchers from other fields to explore rodent neuroimaging.

Recently, progress has been made toward the development of pipelines tailored to the requirements of rodent neuroimagers (Desrosiers-Grégoire et al., 2024;Zerbi et al., 2015). One notable example is the RABIES (Desrosiers-Grégoire et al., 2024) suite, which stands out for its customizability and extensive reporting of quality metrics. However, the maintenance and curation of such open-source consensus pipelines will critically depend on cross-laboratory initiatives aimed at funding and supporting these efforts. Additionally, the impact of commonly used site-harmonization methods employed in human neuroimaging (for example, COMBAT (Orlhac et al., 2022)) has yet to be evaluated within the rodent community. This family of methods needs to be tested and benchmarked against current preprocessing pipelines in rodents.

Furthermore, defining agreed-upon processing and quality assessment guidelines is particularly challenging in the field of fMRI connectivity, where the absence of a ground truth for distinguishing specific from spurious functional connectivity complicates pipeline benchmarking in both humans and rodents. Initial efforts to define empirical data quality metrics have been proposed (Desrosiers-Grégoire et al., 2024), but further benchmarking of pipelines against physiological and anatomical data is needed. To this end, previous studies have compared the topography of functional connectivity networks to the Allen Brain Institute’s mesoscale connectome (Coletta et al., 2020;Grandjean et al., 2017;Whitesell et al., 2021), offering potential anatomical validation for fMRI connectivity networks mapped in conditions of low arousal or anesthesia, where the overlap between structure and function is maximal (Gutierrez-Barragan et al., 2022). As our understanding of the physiological basis of fMRI continues to grow, the extension of cross-validation methods to include complementary readouts, such as multi-site electrophysiology readouts, may help differentiate bona fide functional activity from artifactual or spurious signals (Lake et al., 2020;Rocchi et al., 2022).

## 3 Conclusions

We outlined the exciting potential of rodent whole-brain functional imaging as well as the critical need to address key challenges on the path to a more impactful contribution of this emerging field of research. By fostering collaboration and open science practices, the field can overcome hurdles related to data acquisition consistency, awake imaging complexities, and the extrapolation of results across species (Gutierrez-Barragan et al., 2024;Pagani et al., 2023). Standardization efforts and the development of robust, user-friendly data acquisition and data analysis pipelines will be instrumental in ensuring data quality and reproducibility. Furthermore, adopting multimodal approaches that combine fMRI with other techniques offers great promise for advancing our understanding of brain function. Looking ahead, sustained investment from neuroscience initiatives and funding bodies is crucial to propel this emerging field of translational research forward. Through collaboration and resource sharing, the rodent functional neuroimaging community can unlock the full potential of this powerful tool, significantly advancing our understanding of how brain activity unfolds at the macroscopic level.

## Data Availability

This perspective article does not report original data or findings, nor was any bespoke code written or used in its preparation.

## References

[IMAG.a.12-b1] Bergmann , E. , Zur , G. , Bershadsky , G. , & Kahn , I. ( 2016 ). The organization of mouse and human cortico-hippocampal networks estimated by intrinsic functional connectivity . Cereb Cortex , 26 , 4497 – 4512 . 10.1093/cercor/bhw327 27797832 PMC5193145

[IMAG.a.12-b2] Bernal-Casas , D. , Lee , H. J. , Weitz , A. J. , & Lee , J. H. ( 2017 ). Studying brain circuit function with dynamic causal modeling for optogenetic fMRI . Neuron , 93 , 522 – 532.e525 . 10.1016/j.neuron.2016.12.035 28132829 PMC5472443

[IMAG.a.12-b3] Bertero , A. , Liska , A. , Pagani , M. , Parolisi , R. , Masferrer , M. E. , Gritti , M. , Pedrazzoli , M. , Galbusera , A. , Sarica , A. , Cerasa , A. , Buffelli , M. , Tonini , R. , Buffo , A. , Gross , C. , Pasqualetti , M. , & Gozzi , A. ( 2018 ). Autism-associated 16p11.2 microdeletion impairs prefrontal functional connectivity in mouse and human . Brain , 141 , 2055 – 2065 . 10.1093/brain/awy111 29722793

[IMAG.a.12-b4] Bertolo , A. , Ferrier , J. , Cazzanelli , S. , Diebolt , S. , Tanter , M. , Pezet , S. , Pernot , M. , Osmanski , B.-F. , & Deffieux , T. ( 2023 ). High sensitivity mapping of brain-wide functional networks in awake mice using simultaneous multi-slice fUS imaging . Imaging Neurosci , 1 , 1 – 18 . 10.1162/imag_a_00030 PMC12007538 40799722

[IMAG.a.12-b5] Cabral , J. , Fernandes , F. F. , & Shemesh , N. ( 2023 ). Intrinsic macroscale oscillatory modes driving long range functional connectivity in female rat brains detected by ultrafast fMRI . Nat Commun , 14 , 375 . 10.1038/s41467-023-36025-x 36746938 PMC9902553

[IMAG.a.12-b6] Cerri , D. H. , Albaugh , D. L. , Walton , L. R. , Katz , B. , Wang , T. W. , Chao , T. H. , Zhang , W. , Nonneman , R. J. , Jiang , J. , Lee , S. H. , Etkin , A. , Hall , C. N. , Stuber , G. D. , & Shih , Y. I. ( 2024 ). Distinct neurochemical influences on fMRI response polarity in the striatum . Nat Commun , 15 , 1916 . 10.1038/s41467-024-46088-z 38429266 PMC10907631

[IMAG.a.12-b7] Chan , R. W. , Leong , A. T. L. , Ho , L. C. , Gao , P. P. , Wong , E. C. , Dong , C. M. , Wang , X. , He , J. , Chan , Y. S. , Lim , L. W. , & Wu , E. X. ( 2017 ). Low-frequency hippocampal-cortical activity drives brain-wide resting-state functional MRI connectivity . Proc Natl Acad Sci U S A , 114 , E6972 – E6981 . 10.1073/pnas.1703309114 28760982 PMC5565425

[IMAG.a.12-b8] Chen , X. , Cramer , S. R. , Chan , D. C. Y. , Han , X. , & Zhang , N. ( 2024 ). Sequential deactivation across the hippocampus-thalamus-mPFC pathway during loss of consciousness . Adv Sci , n/a, 2406320 . 10.1101/2024.05.20.594986 PMC11558098 39248326

[IMAG.a.12-b9] Chen , X. , Sobczak , F. , Chen , Y. , Jiang , Y. , Qian , C. , Lu , Z. , Ayata , C. , Logothetis , N. K. , & Yu , X. ( 2019 ). Mapping optogenetically-driven single-vessel fMRI with concurrent neuronal calcium recordings in the rat hippocampus . Nat Commun , 10 , 5239 . 10.1038/s41467-019-12850-x 31748553 PMC6868210

[IMAG.a.12-b10] Chen , Y. , Sobczak , F. , Pais-Roldán , P. , Schwarz , C. , Koretsky , A. P. , & Yu , X. ( 2020 ). Mapping the brain-wide network effects by optogenetic activation of the corpus callosum . Cereb Cortex , 30 , 5885 – 5898 . 10.1093/cercor/bhaa164 32556241 PMC7673479

[IMAG.a.12-b11] Choe , K. Y. , Sanchez , C. F. , Harris , N. G. , Otis , T. S. , & Mathews , P. J. ( 2018 ). Optogenetic fMRI and electrophysiological identification of region-specific connectivity between the cerebellar cortex and forebrain . Neuroimage , 173 , 370 – 383 . 10.1016/j.neuroimage.2018.02.047 29496611 PMC5911204

[IMAG.a.12-b12] Christie , I. N. , Wells , J. A. , Southern , P. , Marina , N. , Kasparov , S. , Gourine , A. V. , & Lythgoe , M. F. ( 2013 ). fMRI response to blue light delivery in the naïve brain: Implications for combined optogenetic fMRI studies . Neuroimage , 66 , 634 – 641 . 10.1016/j.neuroimage.2012.10.074 23128081

[IMAG.a.12-b13] Coletta , L. , Pagani , M. , Whitesell , J. D. , Harris , J. A. , Bernhardt , B. , & Gozzi , A. ( 2020 ). Network structure of the mouse brain connectome with voxel resolution . Sci Adv , 6 , eabb7187 . 10.1126/sciadv.abb7187 33355124 PMC11206455

[IMAG.a.12-b14] Cover , C. G. , Kesner , A. J. , Ukani , S. , Stein , E. A. , Ikemoto , S. , Yang , Y. , & Lu , H. ( 2021 ). Whole brain dynamics during optogenetic self-stimulation of the medial prefrontal cortex in mice . Commun Biol , 4 , 66 . 10.1038/s42003-020-01612-x 33446857 PMC7809041

[IMAG.a.12-b15] Decot , H. K. , Namboodiri , V. M. , Gao , W. , McHenry , J. A. , Jennings , J. H. , Lee , S. H. , Kantak , P. A. , Jill Kao , Y. C. , Das , M. , Witten , I. B. , Deisseroth , K. , Shih , Y. I. , & Stuber , G. D. ( 2017 ). Coordination of brain-wide activity dynamics by dopaminergic neurons . Neuropsychopharmacology , 42 , 615 – 627 . 10.1038/npp.2016.151 27515791 PMC5240174

[IMAG.a.12-b16] Desai , M. , Kahn , I. , Knoblich , U. , Bernstein , J. , Atallah , H. , Yang , A. , Kopell , N. , Buckner , R. L. , Graybiel , A. M. , Moore , C. I. , & Boyden , E. S. ( 2011 ). Mapping brain networks in awake mice using combined optical neural control and fMRI . J Neurophysiol , 105 , 1393 – 1405 . 10.1152/jn.00828.2010 21160013 PMC3074423

[IMAG.a.12-b17] Desrosiers-Grégoire , G. , Devenyi , G. A. , Grandjean , J. , & Chakravarty , M. M. ( 2024 ). A standardized image processing and data quality platform for rodent fMRI . Nat Commun , 15 , 6708 . 10.1038/s41467-024-50826-8 39112455 PMC11306392

[IMAG.a.12-b18] Dinh , T. N. A. , Jung , W. B. , Shim , H.-J. , & Kim , S.-G. ( 2021 ). Characteristics of fMRI responses to visual stimulation in anesthetized vs. awake mice . NeuroImage , 226 , 117542 . 10.1016/j.neuroimage.2020.117542 33186719

[IMAG.a.12-b19] Dvořáková , L. , Stenroos , P. , Salo , R. A. , Paasonen , E. , Tanila , H. , Michaeli , S. , Mangia , S. , Zehnder , T. , Mueggler , T. , Künnecke , B. , Paasonen , J. , & Gröhn , O. ( 2024 ). Whole-brain responses to visual and auditory stimuli in anesthetized and minimally restrained awake mice using quiet zero echo time fMRI . Imaging Neurosci , 2 , 1 – 16 . 10.1162/imag_a_00384 PMC12330383 40800373

[IMAG.a.12-b20] El Hady , A. , Takahashi , D. , Sun , R. , Akinwale , O. , Boyd-Meredith , T. , Zhang , Y. , Charles , A. S. , & Brody , C. D. ( 2024 ). Chronic brain functional ultrasound imaging in freely moving rodents performing cognitive tasks . J Neurosci Methods , 403 , 110033 . 10.1016/j.jneumeth.2023.110033 38056633 PMC10872377

[IMAG.a.12-b21] Ferenczi , E. A. , Zalocusky , K. A. , Liston , C. , Grosenick , L. , Warden , M. R. , Amatya , D. , Katovich , K. , Mehta , H. , Patenaude , B. , Ramakrishnan , C. , Kalanithi , P. , Etkin , A. , Knutson , B. , Glover , G. H. , & Deisseroth , K. ( 2016 ). Prefrontal cortical regulation of brainwide circuit dynamics and reward-related behavior . Science , 351 , aac9698 . 10.1126/science.aac9698 26722001 PMC4772156

[IMAG.a.12-b22] Ferrari , L. , Turrini , G. , Crestan , V. , Bertani , S. , Cristofori , P. , Bifone , A. , & Gozzi , A. ( 2012 ). A robust experimental protocol for pharmacological fMRI in rats and mice . J Neurosci Methods , 204 , 9 – 18 . 10.1016/j.jneumeth.2011.10.020 22068031

[IMAG.a.12-b23] Freeman , R. , & Morris , G. A. ( 2015 ). The Varian story . J Magn Reson , 250 , 80 – 84 . 10.1016/j.jmr.2014.12.001 25532932

[IMAG.a.12-b24] Gent , T. , & Adamantidis , A. ( 2017 ). Anaesthesia and sleep . Clin Transl Neurosci , 1 , 2514183X17726281 . 10.1177/2514183x17726281

[IMAG.a.12-b25] Gil , R. , Valente , M. , & Shemesh , N. ( 2024 ). Rat superior colliculus encodes the transition between static and dynamic vision modes . Nat Commun , 15 , 849 . 10.1038/s41467-024-44934-8 38346973 PMC10861507

[IMAG.a.12-b26] Giorgi , A. , Migliarini , S. , Galbusera , A. , Maddaloni , G. , Mereu , M. , Margiani , G. , Gritti , M. , Landi , S. , Trovato , F. , Bertozzi , S. M. , Armirotti , A. , Ratto , G. M. , De Luca , M. A. , Tonini , R. , Gozzi , A. , & Pasqualetti , M. ( 2017 ). Brain-wide mapping of endogenous serotonergic transmission via chemogenetic fMRI . Cell Rep , 21 , 910 – 918 . 10.1016/j.celrep.2017.09.087 29069598

[IMAG.a.12-b27] Gorgolewski , K. J. , Auer , T. , Calhoun , V. D. , Craddock , R. C. , Das , S. , Duff , E. P. , Flandin , G. , Ghosh , S. S. , Glatard , T. , Halchenko , Y. O. , Handwerker , D. A. , Hanke , M. , Keator , D. , Li , X. , Michael , Z. , Maumet , C. , Nichols , B. N. , Nichols , T. E. , … Poldrack , R. A. ( 2016 ). The brain imaging data structure, a format for organizing and describing outputs of neuroimaging experiments . Sci Data , 3 , 160044 . 10.1038/sdata.2016.44 27326542 PMC4978148

[IMAG.a.12-b28] Gozzi , A. , Ceolin , L. , Schwarz , A. , Reese , T. , Bertani , S. , Crestan , V. , & Bifone , A. ( 2007 ). A multimodality investigation of cerebral hemodynamics and autoregulation in pharmacological MRI . Magn Reson Imaging , 25 , 826 – 833 . 10.1016/j.mri.2007.03.003 17451905

[IMAG.a.12-b29] Gozzi , A. , Jain , A. , Giovannelli , A. , Bertollini , C. , Crestan , V. , Schwarz , A. J. , Tsetsenis , T. , Ragozzino , D. , Gross , C. T. , & Bifone , A. ( 2010 ). A neural switch for active and passive fear . Neuron , 67 , 656 – 666 . 10.1016/j.neuron.2010.07.008 20797541

[IMAG.a.12-b30] Gozzi , A. , & Schwarz , A. J. ( 2016 ). Large-scale functional connectivity networks in the rodent brain . NeuroImage , 127 , 496 – 509 . 10.1016/j.neuroimage.2015.12.017 26706448

[IMAG.a.12-b31] Gozzi , A. , Schwarz , A. , Crestan , V. , & Bifone , A. ( 2008 ). Drug-anaesthetic interaction in phMRI: The case of the psychotomimetic agent phencyclidine . Magn Reson Imaging , 26 , 999 – 1006 . 10.1016/j.mri.2008.01.012 18486387

[IMAG.a.12-b32] Gozzi , A. , & Zerbi , V. ( 2023 ). Modeling brain dysconnectivity in rodents . Biol Psychiatry , 93 , 419 – 429 . 10.1016/j.biopsych.2022.09.008 36517282

[IMAG.a.12-b33] Grandjean , J. , Canella , C. , Anckaerts , C. , Ayranci , G. , Bougacha , S. , Bienert , T. , Buehlmann , D. , Coletta , L. , Gallino , D. , Gass , N. , Garin , C. M. , Nadkarni , N. A. , Hubner , N. S. , Karatas , M. , Komaki , Y. , Kreitz , S. , Mandino , F. , … Mechling , A. E. ( 2020 ). Common functional networks in the mouse brain revealed by multi-centre resting-state fMRI analysis . NeuroImage , 205 , 116278 . 10.1016/j.neuroimage.2019.116278 31614221 PMC7116112

[IMAG.a.12-b34] Grandjean , J. , Corcoba , A. , Kahn , M. C. , Upton , A. L. , Deneris , E. S. , Seifritz , E. , Helmchen , F. , Mann , E. O. , Rudin , M. , & Saab , B. J. ( 2019 ). A brain-wide functional map of the serotonergic responses to acute stress and fluoxetine . Nat Commun , 10 , 350 . 10.1038/s41467-018-08256-w 30664643 PMC6341094

[IMAG.a.12-b35] Grandjean , J. , Desrosiers-Gregoire , G. , Anckaerts , C. , Angeles-Valdez , D. , Ayad , F. , Barrière , D. A. , Blockx , I. , Bortel , A. , Broadwater , M. , & Cardoso , B. M. ( 2023 ). A consensus protocol for functional connectivity analysis in the rat brain . Nat Neurosci , 26 , 673 – 681 . 10.1038/s41593-023-01286-8 36973511 PMC10493189

[IMAG.a.12-b36] Grandjean , J. , Zerbi , V. , Balsters , J. H. , Wenderoth , N. , & Rudin , M. ( 2017 ). Structural basis of large-scale functional connectivity in the mouse . J Neurosci , 37 , 8092 – 8101 . 10.1523/jneurosci.0438-17.2017 28716961 PMC6596781

[IMAG.a.12-b37] Grimm , C. , Duss , S. N. , Privitera , M. , Munn , B. R. , Karalis , N. , Frässle , S. , Wilhelm , M. , Patriarchi , T. , Razansky , D. , Wenderoth , N. , Shine , J. M. , Bohacek , J. , & Zerbi , V. ( 2024 ). Tonic and burst-like locus coeruleus stimulation distinctly shift network activity across the cortical hierarchy . Nat Neurosci , 27 , 2167 – 2177 . 10.1038/s41593-024-01755-8 39284964 PMC11537968

[IMAG.a.12-b38] Grimm , C. , Frässle , S. , Steger , C. , von Ziegler , L. , Sturman , O. , Shemesh , N. , Peleg-Raibstein , D. , Burdakov , D. , Bohacek , J. , Stephan , K. E. , Razansky , D. , Wenderoth , N. , & Zerbi , V. ( 2021 ). Optogenetic activation of striatal D1R and D2R cells differentially engages downstream connected areas beyond the basal ganglia . Cell Rep , 37 , 110161 . 10.1016/j.celrep.2021.110161 34965430

[IMAG.a.12-b39] Grimm , C. , Wenderoth , N. , & Zerbi , V. ( 2022 ). An optimized protocol for assessing changes in mouse whole-brain activity using opto-fMRI . STAR Protocols , 3 , 101761 . 10.1016/j.xpro.2022.101761 36240060 PMC9568887

[IMAG.a.12-b40] Gutierrez-Barragan , D. , Basson , M. A. , Panzeri , S. , & Gozzi , A. ( 2019 ). Infraslow state fluctuations govern spontaneous fMRI network dynamics . Curr Biol , 29 , 2295 – 2306.e2295 . 10.1016/j.cub.2019.06.017 31303490 PMC6657681

[IMAG.a.12-b41] Gutierrez-Barragan , D. , Ramirez , J. S. B. , Panzeri , S. , Xu , T. , & Gozzi , A. ( 2024 ). Evolutionarily conserved fMRI network dynamics in the mouse, macaque, and human brain . Nat Commun , 15 , 8518 . 10.1038/s41467-024-52721-8 39353895 PMC11445567

[IMAG.a.12-b42] Gutierrez-Barragan , D. , Singh , N. A. , Alvino , F. G. , Coletta , L. , Rocchi , F. , De Guzman , E. , Galbusera , A. , Uboldi , M. , Panzeri , S. , & Gozzi , A. ( 2022 ). Unique spatiotemporal fMRI dynamics in the awake mouse brain . Curr Biol , 32 , 631 – 644.e636 . 10.1016/j.cub.2021.12.015 34998465 PMC8837277

[IMAG.a.12-b200] Haas , S. , Bravo , F. , Ionescu , T. M. , Gonzalez-Menendez , I. , Quintanilla-Martinez , L. , Dunkel , G. , Kuebler , L. , Hahn , A. , Lanzenberger , R. , Weigelin , B. , Reischl , G. , Pichler , B. J. , & Herfert , K. ( 2024 ). Functional PET/MRI reveals active inhibition of neuronal activity during optogenetic activation of the nigrostriatal pathway . Sci Adv , 10 , eadn2776. 10.1126/sciadv.adn2776 PMC11506239 39454014

[IMAG.a.12-b43] Hamada , H. T. , Abe , Y. , Takata , N. , Taira , M. , Tanaka , K. F. , & Doya , K. ( 2024 ). Optogenetic activation of dorsal raphe serotonin neurons induces brain-wide activation . Nat Commun , 15 , 4152 . 10.1038/s41467-024-48489-6 38755120 PMC11099070

[IMAG.a.12-b44] Han , X. , Cramer , S. R. , & Zhang , N. ( 2022 ). Deriving causal relationships in resting-state functional connectivity using SSFO-based optogenetic fMRI . J Neural Eng , 19 . 10.1088/1741-2552/ac9d66 PMC9681600 36301683

[IMAG.a.12-b45] Helbing , C. , Brocka , M. , Arboit , A. , Lippert , M. T. , & Angenstein , F. ( 2024 ). Chemogenetic inhibition of dopaminergic neurons reduces stimulus-induced dopamine release, thereby altering the hemodynamic response function in the prefrontal cortex . Imaging Neurosci , 2 , 1 – 16 . 10.1162/imag_a_00200 PMC12272248 40800412

[IMAG.a.12-b46] Hike , D. , Liu , X. , Xie , Z. , Zhang , B. , Choi , S. , Zhou , X. A. , Liu , A. , Murstein , A. , Jiang , Y. , Devor , A. , & Yu , X. ( 2024 ). High-resolution awake mouse fMRI at 14 Tesla . bioRxiv , 2023.2012.2008.570803. 10.7554/elife.95528.1 PMC11717365 39786364

[IMAG.a.12-b150] Idiyatullin , D. , Corum , C. A. , & Garwood , M. ( 2015 ). Multi-band-SWIFT . Journal of Magnetic Resonance , 251 , 19 – 25 . https://doi.org/10.1016/j.jmr.2014.11.014 25557859 10.1016/j.jmr.2014.11.014 PMC4329075

[IMAG.a.12-b47] Ioanas , H. I. , Saab , B. J. , & Rudin , M. ( 2022 ). Whole-brain opto-fMRI map of mouse VTA dopaminergic activation reflects structural projections with small but significant deviations . Transl Psychiatry , 12 , 60 . 10.1038/s41398-022-01812-5 35165257 PMC8844000

[IMAG.a.12-b201] Ionescu , T. , Amend , M. , Hafiz , R. , Maurer , A. , Biswal , B. , Wehrl , H. , & Herfert , K. ( 2025 ). Mapping serotonergic dynamics using drug-modulated molecular connectivity in rats . eLife , 13 , RP97864. 10.7554/eLife.97864.3 PMC12080997 40372776

[IMAG.a.12-b48] Iordanova , B. , Vazquez , A. L. , Poplawsky , A. J. , Fukuda , M. , & Kim , S. G. ( 2015 ). Neural and hemodynamic responses to optogenetic and sensory stimulation in the rat somatosensory cortex . J Cereb Blood Flow Metab , 35 , 922 – 932 . 10.1038/jcbfm.2015.10 25669905 PMC4640245

[IMAG.a.12-b49] Jung , W. B. , Im , G. H. , Jiang , H. , & Kim , S.-G. ( 2021 ). Early fMRI responses to somatosensory and optogenetic stimulation reflect neural information flow . Proc Natl Acad Sci , 118 , e2023265118 . 10.1073/pnas.2023265118 33836602 PMC7980397

[IMAG.a.12-b50] Jung , W. B. , Jiang , H. , Lee , S. , & Kim , S. G. ( 2022 ). Dissection of brain-wide resting-state and functional somatosensory circuits by fMRI with optogenetic silencing . Proc Natl Acad Sci U S A , 119 , e2113313119 . 10.1073/pnas.2113313119 35042795 PMC8795561

[IMAG.a.12-b51] Kahn , I. , Desai , M. , Knoblich , U. , Bernstein , J. , Henninger , M. , Graybiel , A. M. , Boyden , E. S. , Buckner , R. L. , & Moore , C. I. ( 2011 ). Characterization of the functional MRI response temporal linearity via optical control of neocortical pyramidal neurons . J Neurosci , 31 , 15086 – 15091 . 10.1523/jneurosci.0007-11.2011 22016542 PMC3225054

[IMAG.a.12-b52] Kahn , I. , Knoblich , U. , Desai , M. , Bernstein , J. , Graybiel , A. M. , Boyden , E. S. , Buckner , R. L. , & Moore , C. I. ( 2013 ). Optogenetic drive of neocortical pyramidal neurons generates fMRI signals that are correlated with spiking activity . Brain Res , 1511 , 33 – 45 . 10.1016/j.brainres.2013.03.011 23523914 PMC3766586

[IMAG.a.12-b53] Kargl , D. , Kaczanowska , J. , Ulonska , S. , Groessl , F. , Piszczek , L. , Lazovic , J. , Buehler , K. , & Haubensak , W. ( 2020 ). The amygdala instructs insular feedback for affective learning . Elife , 9 , e60336 . 10.7554/elife.60336 33216712 PMC7679142

[IMAG.a.12-b54] Kim , S. , Moon , H. S. , Vo , T. T. , Kim , C. H. , Im , G. H. , Lee , S. , Choi , M. , & Kim , S. G. ( 2023 ). Whole-brain mapping of effective connectivity by fMRI with cortex-wide patterned optogenetics . Neuron , 111 , 1732 – 1747.e1736 . 10.1016/j.neuron.2023.03.002 37001524

[IMAG.a.12-b55] Kovács , P. , Beloate , L. N. , & Zhang , N. ( 2024 ). Perturbing cortical networks: In vivo electrophysiological consequences of pan-neuronal chemogenetic manipulations using deschloroclozapine . Front Neurosci , 18 , 1396978 . 10.3389/fnins.2024.1396978 38726028 PMC11079238

[IMAG.a.12-b56] Lake , E. M. R. , Ge , X. , Shen , X. , Herman , P. , Hyder , F. , Cardin , J. A. , Higley , M. J. , Scheinost , D. , Papademetris , X. , Crair , M. C. , & Constable , R. T. ( 2020 ). Simultaneous cortex-wide fluorescence Ca(2+) imaging and whole-brain fMRI . Nat Methods , 17 , 1262 – 1271 . 10.1038/s41592-020-00984-6 33139894 PMC7704940

[IMAG.a.12-b57] Lee , H. J. , Weitz , A. J. , Bernal-Casas , D. , Duffy , B. A. , Choy , M. , Kravitz , A. V. , Kreitzer , A. C. , & Lee , J. H. ( 2016 ). Activation of direct and indirect pathway medium spiny neurons drives distinct brain-wide responses . Neuron , 91 , 412 – 424 . 10.1016/j.neuron.2016.06.010 27373834 PMC5528162

[IMAG.a.12-b58] Lee , J. H. , Durand , R. , Gradinaru , V. , Zhang , F. , Goshen , I. , Kim , D. S. , Fenno , L. E. , Ramakrishnan , C. , & Deisseroth , K. ( 2010 ). Global and local fMRI signals driven by neurons defined optogenetically by type and wiring . Nature , 465 , 788 – 792 . 10.1038/nature09108 20473285 PMC3177305

[IMAG.a.12-b59] Lee , J. Y. , You , T. , Lee , C. H. , Im , G. H. , Seo , H. , Woo , C. W. , & Kim , S. G. ( 2022 ). Role of anterior cingulate cortex inputs to periaqueductal gray for pain avoidance . Curr Biol , 32 , 2834 – 2847.e2835 . 10.1016/j.cub.2022.04.090 35609604

[IMAG.a.12-b204] Lee , S. , Jung , W. B. , Moon , H. , Im , G. H. , Noh , Y. W. , Shin , W. , Kim , Y. G. , Yi , J. H. , Hong , S, J. , Jung , Y. , Ahn , S. , Kim , S. G. , & Kim , E. ( 2024 ). Anterior cingulate cortex-related functional hyperconnectivity underlies sensory hypersensitivity in *Grin2b* -mutant mice . Mol Psychiatry , 29 , 3195 – 3207 . 10.1038/s41380-024-02572-y 38704508 PMC11449790

[IMAG.a.12-b205] Lehto , L. J. , Idiyatullin , D. , Zhang , J. , Utecht , L. , Adriany , G. , Garwood , M. , Gröhn , O. , Michaeli , S. , Mangia , S. ( 2017 ). MB-SWIFT functional MRI during deep brain stimulation in rats . NeuroImage , 159 , 443 – 448 . 10.1016/j.neuroimage.2017.08.012 28797739 PMC5671345

[IMAG.a.12-b60] Leong , A. T. , Chan , R. W. , Gao , P. P. , Chan , Y. S. , Tsia , K. K. , Yung , W. H. , & Wu , E. X. ( 2016 ). Long-range projections coordinate distributed brain-wide neural activity with a specific spatiotemporal profile . Proc Natl Acad Sci U S A , 113 , E8306 – e8315 . 10.1073/pnas.1616361113 27930323 PMC5187697

[IMAG.a.12-b61] Leong , A. T. L. , Wang , X. , Wong , E. C. , Dong , C. M. , & Wu , E. X. ( 2021 ). Neural activity temporal pattern dictates long-range propagation targets . Neuroimage , 235 , 118032 . 10.1016/j.neuroimage.2021.118032 33836268

[IMAG.a.12-b62] Li , N. , Downey , J. E. , Bar-Shir , A. , Gilad , A. A. , Walczak , P. , Kim , H. , Joel , S. E. , Pekar , J. J. , Thakor , N. V. , & Pelled , G. ( 2011 ). Optogenetic-guided cortical plasticity after nerve injury . Proc Natl Acad Sci U S A , 108 , 8838 – 8843 . 10.1073/pnas.1100815108 21555573 PMC3102379

[IMAG.a.12-b63] Li , Y. , Lee , S. H. , Yu , C. , Hsu , L. M. , Wang , T. W. , Do , K. , Kim , H. J. , Shih , Y. I. , & Grill , W. M. ( 2024 ). Optogenetic fMRI reveals therapeutic circuits of subthalamic nucleus deep brain stimulation . bioRxiv . 10.1101/2024.02.22.581627 PMC11364984 39096961

[IMAG.a.12-b64] Li , Z. , Athwal , D. , Lee , H. L. , Sah , P. , Opazo , P. , & Chuang , K. H. ( 2023 ). Locating causal hubs of memory consolidation in spontaneous brain network in male mice . Nat Commun , 14 , 5399 . 10.1038/s41467-023-41024-z 37669938 PMC10480429

[IMAG.a.12-b65] Liang , Z. , King , J. , & Zhang , N. ( 2011 ). Uncovering intrinsic connectional architecture of functional networks in awake rat brain . J Neurosci , 31 , 3776 – 3783 . 10.1523/jneurosci.4557-10.2011 21389232 PMC3073070

[IMAG.a.12-b66] Liang , Z. , King , J. , & Zhang , N. ( 2012 ). Anticorrelated resting-state functional connectivity in awake rat brain . NeuroImage , 59 , 1190 – 1199 . 10.1016/j.neuroimage.2011.08.009 21864689 PMC3230741

[IMAG.a.12-b67] Liang , Z. , Watson , G. D. , Alloway , K. D. , Lee , G. , Neuberger , T. , & Zhang , N. ( 2015 ). Mapping the functional network of medial prefrontal cortex by combining optogenetics and fMRI in awake rats . Neuroimage , 117 , 114 – 123 . 10.1016/j.neuroimage.2015.05.036 26002727 PMC4512884

[IMAG.a.12-b202] Ljungberg , E. , Damestani , N. L. , Wood , T. C. , Lythgoe , D. J. , Zelaya , F. , Williams , S. C. R. , Solana , A. B. , Barker , G. J. , & Wiesinger , F. ( 2021 ). Silent zero TE MR neuroimaging: Current state-of-the-art and future directions . Prog Nucl Magn Reson Spectrosc , 123 , 73 – 93 . 10.1016/j.pnmrs.2021.03.002 34078538 PMC7616227

[IMAG.a.12-b68] MacKinnon , M. J. , Song , S. , Chao , T.-H. H. , Hsu , L.-M. , Albert , S. T. , Ma , Y. , Shnitko , T. A. , Wang , T.-W. W. , Nonneman , R. J. , Freeman , C. D. , Ozarkar , S. S. , Emir , U. E. , Shen , M. D. , Philpot , B. D. , Hantman , A. W. , Lee , S.-H. , Chang , W.-T. , & Shih , Y.-Y. I. ( 2025 ). SORDINO for silent, sensitive, specific, and artifact-resisting fMRI in awake behaving mice . bioRxiv , 2025.2003.2010.642406. 10.1101/2025.03.10.642406 42717250

[IMAG.a.12-b203] Madio , D. P. , & Lowe , I. J. ( 1995 ). Ultra-fast imaging using low flip angles and FIDs . Magn Reson Med , 34 , 525 – 529 . 10.1002/mrm.1910340407 8524019

[IMAG.a.12-b69] Mandino , F. , Vrooman , R. M. , Foo , H. E. , Yeow , L. Y. , Bolton , T. A. W. , Salvan , P. , Teoh , C. L. , Lee , C. Y. , Beauchamp , A. , Luo , S. , Bi , R. , Zhang , J. , Lim , G. H. T. , Low , N. , Sallet , J. , Gigg , J. , Lerch , J. P. , Mars , R. B. , Olivo , M. , Fu , Y. , & Grandjean , J. ( 2022 ). A triple-network organization for the mouse brain . Mol Psychiatry , 27 , 865 – 872 . 10.1038/s41380-021-01298-5 34650202 PMC9054663

[IMAG.a.12-b70] Mandino , F. , Vujic , S. , Grandjean , J. , & Lake , E. M. R. ( 2024 ). Where do we stand on fMRI in awake mice? Cereb Cortex , 34 , bhad478 . 10.1093/cercor/bhad478 38100331 PMC10793583

[IMAG.a.12-b71] Markicevic , M. , Sturman , O. , Bohacek , J. , Rudin , M. , Zerbi , V. , Fulcher , B. D. , & Wenderoth , N. ( 2023 ). Neuromodulation of striatal D1 cells shapes BOLD fluctuations in anatomically connected thalamic and cortical regions . Elife , 12 , e78620 . 10.7554/elife.78620 37824184 PMC10569790

[IMAG.a.12-b72] Menon , V. , Cerri , D. , Lee , B. , Yuan , R. , Lee , S. H. , & Shih , Y. I. ( 2023 ). Optogenetic stimulation of anterior insular cortex neurons in male rats reveals causal mechanisms underlying suppression of the default mode network by the salience network . Nat Commun , 14 , 866 . 10.1038/s41467-023-36616-8 36797303 PMC9935890

[IMAG.a.12-b73] Meyer-Baese , L. , Morrissette , A. E. , Wang , Y. , Le Chatelier , B. , Borden , P. Y. , Keilholz , S. D. , Stanley , G. B. , & Jaeger , D. ( 2024 ). Cortical networks relating to arousal are differentially coupled to neural activity and hemodynamics . J Neurosci , 44 , e0298232024 . 10.1101/2022.12.01.518759 38769007 PMC11209646

[IMAG.a.12-b206] Millevert , C. , Vidas-Guscic , N. , Vanherp , L. , Jonckers , E. , Verhoye , M. , Staelens , S. , Bertoglio , D. , & Weckhuysen , S. ( 2023 ). Resting-state functional MRI and PET imaging as noninvasive tools to study (ab)normal neurodevelopment in humans and rodents . J Neurosci , 43 , 8275 – 8293 . 10.1523/JNEUROSCI.1043-23.2023 38073598 PMC10711730

[IMAG.a.12-b74] Moon , H. S. , Jiang , H. , Vo , T. T. , Jung , W. B. , Vazquez , A. L. , & Kim , S. G. ( 2021 ). Contribution of excitatory and inhibitory neuronal activity to BOLD fMRI . Cereb Cortex , 31 , 4053 – 4067 . 10.1093/cercor/bhab068 33895810 PMC8328221

[IMAG.a.12-b75] Nakamura , Y. , Nakamura , Y. , Pelosi , A. , Djemai , B. , Debacker , C. , Hervé , D. , Girault , J.-A. , & Tsurugizawa , T. ( 2020 ). fMRI detects bilateral brain network activation following unilateral chemogenetic activation of direct striatal projection neurons . Neuroimage , 220 , 117079 . 10.1016/j.neuroimage.2020.117079 32585345

[IMAG.a.12-b76] Nasrallah , F. A. , Tay , H. C. , & Chuang , K. H. ( 2014 ). Detection of functional connectivity in the resting mouse brain . NeuroImage , 86 , 417 – 424 . 10.1016/j.neuroimage.2013.10.025 24157920

[IMAG.a.12-b77] Orlhac , F. , Eertink , J. J. , Cottereau , A. S. , Zijlstra , J. M. , Thieblemont , C. , Meignan , M. , Boellaard , R. , & Buvat , I. ( 2022 ). A guide to ComBat harmonization of imaging biomarkers in multicenter studies . J Nucl Med , 63 , 172 – 179 . 10.2967/jnumed.121.262464 34531263 PMC8805779

[IMAG.a.12-b78] Oyarzabal , E. A. , Hsu , L.-M. , Das , M. , Chao , T.-H. H. , Zhou , J. , Song , S. , Zhang , W. , Smith , K. G. , Sciolino , N. R. , Evsyukova , I. Y. , Yuan , H. , Lee , S.-H. , Cui , G. , Jensen , P. , & Shih , Y.-Y. I. ( 2022 ). Chemogenetic stimulation of tonic locus coeruleus activity strengthens the default mode network . Sci Adv , 8 , eabm9898 . 10.1126/sciadv.abm9898 35486721 PMC9054017

[IMAG.a.12-b79] Paasonen , J. , Laakso , H. , Pirttimaki , T. , Stenroos , P. , Salo , R. A. , Zhurakovskaya , E. , Lehto , L. J. , Tanila , H. , Garwood , M. , Michaeli , S. , Idiyatullin , D. , Mangia , S. , & Grohn , O. ( 2020 ). Multi-band SWIFT enables quiet and artefact-free EEG-fMRI and awake fMRI studies in rat . NeuroImage , 206 , 116338 . 10.1016/j.neuroimage.2019.116338 31730923 PMC7008094

[IMAG.a.12-b80] Pagani , M. , Gutierrez-Barragan , D. , de Guzman , A. E. , Xu , T. , & Gozzi , A. ( 2023 ). Mapping and comparing fMRI connectivity networks across species . Commun Biol , 6 , 1238 . 10.1038/s42003-023-05629-w 38062107 PMC10703935

[IMAG.a.12-b81] Peeters , L. M. , Hinz , R. , Detrez , J. R. , Missault , S. , De Vos , W. H. , Verhoye , M. , Van der Linden , A. , & Keliris , G. A. ( 2020 ). Chemogenetic silencing of neurons in the mouse anterior cingulate area modulates neuronal activity and functional connectivity . NeuroImage , 220 , 117088 . 10.1016/j.neuroimage.2020.117088 32592851

[IMAG.a.12-b82] Percie du Sert , N. , Hurst , V. , Ahluwalia , A. , Alam , S. , Avey , M. T. , Baker , M. , Browne , W. J. , Clark , A. , Cuthill , I. C. , Dirnagl , U. , Emerson , M. , Garner , P. , Holgate , S. T. , Howells , D. W. , Karp , N. A. , Lazic , S. E. , Lidster , K. , MacCallum , C. J. , Macleod , M. , … Wurbel , H. ( 2020 ). The ARRIVE guidelines 2.0: Updated guidelines for reporting animal research . Exp Physiol , 105 , 1459 – 1466 . 10.1113/ep088870 32666546 PMC7610926

[IMAG.a.12-b83] Pisauro , M. A. , Benucci , A. , & Carandini , M. ( 2016 ). Local and global contributions to hemodynamic activity in mouse cortex . J Neurophysiol , 115 , 2931 – 2936 . 10.1152/jn.00125.2016 26984421 PMC4922613

[IMAG.a.12-b84] Ramos-Cabrer , P. , Weber , R. , Wiedermann , D. , & Hoehn , M. ( 2005 ). Continuous noninvasive monitoring of transcutaneous blood gases for a stable and persistent BOLD contrast in fMRI studies in the rat . NMR Biomed , 18 , 440 – 446 . 10.1002/nbm.978 16158460

[IMAG.a.12-b85] Reimann , H. M. , & Niendorf , T. ( 2020 ). The (Un)Conscious mouse as a model for human brain functions: Key principles of anesthesia and their impact on translational neuroimaging . Front Syst Neurosci , 14 , 8 . 10.3389/fnsys.2020.00008 32508601 PMC7248373

[IMAG.a.12-b86] Reimann , H. M. , Todiras , M. , Hodge , R. , Huelnhagen , T. , Millward , J. M. , Turner , R. , Seeliger , E. , Bader , M. , Pohlmann , A. , & Niendorf , T. ( 2018 ). Somatosensory BOLD fMRI reveals close link between salient blood pressure changes and the murine neuromatrix . NeuroImage , 172 , 562 – 574 . 10.1016/j.neuroimage.2018.02.002 29421323

[IMAG.a.12-b87] Rocchi , F. , Canella , C. , Noei , S. , Gutierrez-Barragan , D. , Coletta , L. , Galbusera , A. , Stuefer , A. , Vassanelli , S. , Pasqualetti , M. , Iurilli , G. , Panzeri , S. , & Gozzi , A. ( 2022 ). Increased fMRI connectivity upon chemogenetic inhibition of the mouse prefrontal cortex . Nat Commun , 13 , 1056 . 10.1038/s41467-022-28591-3 35217677 PMC8881459

[IMAG.a.12-b88] Roelofs , T. J. M. , Verharen , J. P. H. , van Tilborg , G. A. F. , Boekhoudt , L. , van der Toorn , A. , de Jong , J. W. , Luijendijk , M. C. M. , Otte , W. M. , Adan , R. A. H. , & Dijkhuizen , R. M. ( 2017 ). A novel approach to map induced activation of neuronal networks using chemogenetics and functional neuroimaging in rats: A proof-of-concept study on the mesocorticolimbic system . Neuroimage , 156 , 109 – 118 . 10.1016/j.neuroimage.2017.05.021 28502844

[IMAG.a.12-b89] Rungta , R. L. , Osmanski , B. F. , Boido , D. , Tanter , M. , & Charpak , S. ( 2017 ). Light controls cerebral blood flow in naive animals . Nat Commun , 8 , 14191 . 10.1038/ncomms14191 28139643 PMC5290324

[IMAG.a.12-b90] Ryali , S. , Shih , Y. Y. , Chen , T. , Kochalka , J. , Albaugh , D. , Fang , Z. , Supekar , K. , Lee , J. H. , & Menon , V. ( 2016 ). Combining optogenetic stimulation and fMRI to validate a multivariate dynamical systems model for estimating causal brain interactions . Neuroimage , 132 , 398 – 405 . 10.1016/j.neuroimage.2016.02.067 26934644 PMC4851892

[IMAG.a.12-b207] Sabatini , B. L. , & Tian , L. ( 2020 ). Imaging neurotransmitter and neuromodulator dynamics in vivo with genetically encoded indicators . Neuron , 108 , 17 – 32 . 10.1016/j.neuron.2020.09.036 33058762

[IMAG.a.12-b91] Salvan , P. , Lazari , A. , Vidaurre , D. , Mandino , F. , Johansen-Berg , H. , & Grandjean , J. ( 2021 ). Frequency modulation of entorhinal cortex neuronal activity drives distinct frequency-dependent states of brain-wide dynamics . Cell Rep , 37 , 109954 . 10.1016/j.celrep.2021.109954 34731612 PMC8609366

[IMAG.a.12-b92] Sanda , P. , Hlinka , J. , van den Berg , M. , Skoch , A. , Bazhenov , M. , Keliris , G. A. , & Krishnan , G. P. ( 2024 ). Cholinergic modulation supports dynamic switching of resting state networks through selective DMN suppression . PLoS Comput Biol , 20 , e1012099 . 10.1371/journal.pcbi.1012099 38843298 PMC11185486

[IMAG.a.12-b93] Schmid , F. , Wachsmuth , L. , Albers , F. , Schwalm , M. , Stroh , A. , & Faber , C. ( 2017 ). True and apparent optogenetic BOLD fMRI signals . Magn Reson Med , 77 , 126 – 136 . 10.1002/mrm.26095 26778283

[IMAG.a.12-b94] Sforazzini , F. , Schwarz , A. J. , Galbusera , A. , Bifone , A. , & Gozzi , A. ( 2014 ). Distributed BOLD and CBV-weighted resting-state networks in the mouse brain . NeuroImage , 87 , 403 – 415 . 10.1016/j.neuroimage.2013.09.050 24080504

[IMAG.a.12-b95] Siddiqi , S. H. , Kording , K. P. , Parvizi , J. , & Fox , M. D. ( 2022 ). Causal mapping of human brain function . Nat Rev Neurosci , 23 , 361 – 375 . 10.1038/s41583-022-00583-8 35444305 PMC9387758

[IMAG.a.12-b96] Sirmpilatze , N. , Mylius , J. , Ortiz-Rios , M. , Baudewig , J. , Paasonen , J. , Golkowski , D. , Ranft , A. , Ilg , R. , Grohn , O. , & Boretius , S. ( 2022 ). Spatial signatures of anesthesia-induced burst-suppression differ between primates and rodents . eLife , 11 , e74813 . 10.7554/elife.74813 35607889 PMC9129882

[IMAG.a.12-b97] Sonner , J. M. , Gong , D. , & Eger , E. I. ( 2000 ). Naturally occurring variability in anesthetic potency among inbred mouse strains . Anesth Analg , 91 , 720 – 726 . 10.1213/00000539-200009000-00042 10960407

[IMAG.a.12-b98] Stringer , C. , Pachitariu , M. , Steinmetz , N. , Reddy , C. B. , Carandini , M. , & Harris , K. D. ( 2019 ). Spontaneous behaviors drive multidimensional, brainwide activity . Science , 364 , 255 . 10.1126/science.aav7893 31000656 PMC6525101

[IMAG.a.12-b99] Takata , N. , Sugiura , Y. , Yoshida , K. , Koizumi , M. , Hiroshi , N. , Honda , K. , Yano , R. , Komaki , Y. , Matsui , K. , Suematsu , M. , Mimura , M. , Okano , H. , & Tanaka , K. F. ( 2018 ). Optogenetic astrocyte activation evokes BOLD fMRI response with oxygen consumption without neuronal activity modulation . Glia , 66 , 2013 – 2023 . 10.1002/glia.23454 29845643

[IMAG.a.12-b100] Tu , W. , & Zhang , N. ( 2022 ). Neural underpinning of a respiration-associated resting-state fMRI network . eLife , 11 , e81555 . 10.7554/elife.81555 36263940 PMC9645809

[IMAG.a.12-b101] Vo , Thanh T. , Im , Geun H. , Han , K. , Suh , M. , Drew , Patrick J. , & Kim , S.-G. ( 2023 ). Parvalbumin interneuron activity drives fast inhibition-induced vasoconstriction followed by slow substance P-mediated vasodilation . Proc Natl Acad Sci , 120 , e2220777120 . 10.1073/pnas.2220777120 37098063 PMC10161000

[IMAG.a.12-b102] Wang , X. , Leong , A. T. L. , Tan , S. Z. K. , Wong , E. C. , Liu , Y. , Lim , L.-W. , & Wu , E. X. ( 2023 ). Functional MRI reveals brain-wide actions of thalamically-initiated oscillatory activities on associative memory consolidation . Nat Commun , 14 , 2195 . 10.1038/s41467-023-37682-8 37069169 PMC10110623

[IMAG.a.12-b208] Wehrl , H. F. , Hossain , M. , Lankes , K. , Liu , C.-C. , Bezrukov , I. , Martirosian , P. , Schick , F. , Reischl , G. , & Pichler , B. J. ( 2013 ). Simultaneous PET-MRI reveals brain function in activated and resting state on metabolic, hemodynamic and multiple temporal scales . Nat Med , 19 , 1184 – 1189 . 10.1038/nm.3290 23975025

[IMAG.a.12-b103] Wei , X. , Centeno , M. V. , Ren , W. , Borruto , A. M. , Procissi , D. , Xu , T. , Jabakhanji , R. , Mao , Z. , Kim , H. , Li , Y. , Yang , Y. , Gutruf , P. , Rogers , J. A. , Surmeier , D. J. , Radulovic , J. , Liu , X. , Martina , M. , & Apkarian , A. V. ( 2021 ). Activation of the dorsal, but not the ventral, hippocampus relieves neuropathic pain in rodents . Pain , 162 , 2865 – 2880 . 10.1097/j.pain.0000000000002279 34160168 PMC8464622

[IMAG.a.12-b209] Weiger , M. , & Pruessmann , K. P. ( 2019 ). Short-T _2_ MRI: Principles and recent advances . Prog Nucl Magn Reson Spectrosc , 114–115 , 237 – 270 . 10.1016/j.pnmrs.2019.07.001 31779882

[IMAG.a.12-b104] Weitz , A. J. , Fang , Z. , Lee , H. J. , Fisher , R. S. , Smith , W. C. , Choy , M. , Liu , J. , Lin , P. , Rosenberg , M. , & Lee , J. H. ( 2015 ). Optogenetic fMRI reveals distinct, frequency-dependent networks recruited by dorsal and intermediate hippocampus stimulations . Neuroimage , 107 , 229 – 241 . 10.1016/j.neuroimage.2014.10.039 25462689 PMC4409430

[IMAG.a.12-b105] Whitesell , J. D. , Liska , A. , Coletta , L. , Hirokawa , K. E. , Bohn , P. , Williford , A. , Groblewski , P. A. , Graddis , N. , Kuan , L. , Knox , J. E. , Ho , A. , Wakeman , W. , Nicovich , P. R. , Nguyen , T. N. , van Velthoven , C. T. J. , Garren , E. , Fong , O. , Naeemi , M. , … Harris , J. A. ( 2021 ). Regional, layer, and cell-type-specific connectivity of the mouse default mode network . Neuron , 109 , 545 – 559.e548 . 10.1016/j.neuron.2020.11.011 33290731 PMC8150331

[IMAG.a.12-b106] Winder , A. T. , Echagarruga , C. , Zhang , Q. , & Drew , P. J. ( 2017 ). Weak correlations between hemodynamic signals and ongoing neural activity during the resting state . Nat Neurosci , 20 , 1761 – 1769 . 10.1038/s41593-017-0007-y 29184204 PMC5816345

[IMAG.a.12-b107] Yu , X. , He , Y. , Wang , M. , Merkle , H. , Dodd , S. J. , Silva , A. C. , & Koretsky , A. P. ( 2016 ). Sensory and optogenetically driven single-vessel fMRI . Nat Methods , 13 , 337 – 340 . 10.1038/nmeth.3765 26855362 PMC6298439

[IMAG.a.12-b108] Zerbi , V. , Floriou-Servou , A. , Markicevic , M. , Vermeiren , Y. , Sturman , O. , Privitera , M. , von Ziegler , L. , Ferrari , K. D. , Weber , B. , De Deyn , P. P. , Wenderoth , N. , & Bohacek , J. ( 2019 ). Rapid reconfiguration of the functional connectome after chemogenetic locus coeruleus activation . Neuron , 103 , 702 – 718.e705 . 10.1016/j.neuron.2019.05.034 31227310

[IMAG.a.12-b109] Zerbi , V. , Grandjean , J. , Rudin , M. , & Wenderoth , N. ( 2015 ). Mapping the mouse brain with rs-fMRI: An optimized pipeline for functional network identification . NeuroImage , 123 , 11 – 21 . 10.1016/j.neuroimage.2015.07.090 26296501

[IMAG.a.12-b110] Zerbi , V. , Pagani , M. , Markicevic , M. , Matteoli , M. , Pozzi , D. , Fagiolini , M. , Bozzi , Y. , Galbusera , A. , Scattoni , M. L. , Provenzano , G. , Banerjee , A. , Helmchen , F. , Basson , M. A. , Ellegood , J. , Lerch , J. P. , Rudin , M. , Gozzi , A. , & Wenderoth , N. ( 2021 ). Brain mapping across 16 autism mouse models reveals a spectrum of functional connectivity subtypes . Mol Psychiatry , 26 , 7610 – 7620 . 10.1038/s41380-021-01245-4 34381171 PMC8873017

[IMAG.a.12-b111] Zou , Y. , Tong , C. , Peng , W. , Qiu , Y. , Li , J. , Xia , Y. , Pei , M. , Zhang , K. , Li , W. , Xu , M. , & Liang , Z. ( 2024 ). Cell-type-specific optogenetic fMRI on basal forebrain reveals functional network basis of behavioral preference . Neuron , 112 , 1342 – 1357.e1346 . 10.1016/j.neuron.2024.01.017 38359827

